# Prediction and classification of obesity risk based on a hybrid metaheuristic machine learning approach

**DOI:** 10.3389/fdata.2024.1469981

**Published:** 2024-09-30

**Authors:** Zarindokht Helforoush, Hossein Sayyad

**Affiliations:** ^1^Department of Mathematics and Systems Engineering, Florida Institute of Technology, Melbourne, FL, United States; ^2^Independent Researcher, Melbourne, FL, United States

**Keywords:** Artificial Neural Network, Particle Swarm Optimization, hyperparameter tuning, obesity classification, public health, metaheuristic algorithms

## Abstract

**Introduction:**

As the global prevalence of obesity continues to rise, it has become a major public health concern requiring more accurate prediction methods. Traditional regression models often fail to capture the complex interactions between genetic, environmental, and behavioral factors contributing to obesity.

**Methods:**

This study explores the potential of machine-learning techniques to improve obesity risk prediction. Various supervised learning algorithms, including the novel ANN-PSO hybrid model, were applied following comprehensive data preprocessing and evaluation.

**Results:**

The proposed ANN-PSO model achieved a remarkable accuracy rate of 92%, outperforming traditional regression methods. SHAP was employed to analyze feature importance, offering deeper insights into the influence of various factors on obesity risk.

**Discussion:**

The findings highlight the transformative role of advanced machine-learning models in public health research, offering a pathway for personalized healthcare interventions. By providing detailed obesity risk profiles, these models enable healthcare providers to tailor prevention and treatment strategies to individual needs. The results underscore the need to integrate innovative machine-learning approaches into global public health efforts to combat the growing obesity epidemic.

## 1 Introduction

As a complicated and unsolvable public health and medical issue, obesity has spread around the world and has serious adverse effects on both health and the economy (Ward et al., 2019). This condition, which manifests as related medical and psychological conditions, places a significant burden on one's health and social life. World Health Organization (WHO) defines obesity as an excess of adiposity, which is a build-up of body fat that is harmful to one's health (Word Health Organization, 2023). In order to operationalize this definition, body mass index (BMI), a widely utilized measure of body fat, is used to categorize obesity. More specifically, according to WHO guidelines, individuals are classified as obese if their BMI exceeds 30 kg/m^2^. Obesity patients endure persistent discrimination and stigma, which raises the risk of illness and death (Afshin et al., 2017). In addition to the psychological consequences, obesity imposes substantial financial burdens on healthcare systems and entire communities (Busebee et al., 2023; Ward et al., 2019). The prevalence of obesity has nearly doubled globally since 1980. According to James et al. (2001), presently, there exists a population of more than 200 million adult males and ~300 million adult females who are afflicted with obesity. Furthermore, estimates indicate that by 2030, obesity will affect roughly one in every two adults in the US (Ward et al., 2019). Various ailments, including hypertension (elevated blood pressure), cardiovascular disease, diabetes, stroke, and different forms of cancer, are linked to this increase in the prevalence of obesity (Pi-Sunyer, 1999). Obesity is also associated with psychological effects, joint strain, and hormonal imbalances. Research has repeatedly shown that people with BMI typically live shorter lives (Berraho et al., 2010; Flegal et al., 2007). Obesity greatly increases the risk of severe COVID-19, as it raises the chances of negative outcomes such as admission to the hospital or the Intensive Care Unit (ICU) and death (Arulanandam et al., 2023; Singh et al., 2022; Steenblock et al., 2022). This association can be attributed to various metabolic effects, inflammation, and compromised lung function. Regression models have historically been the main tool researchers use to assess the risk of obesity, with a preference for linear modeling approaches. However, the pursuit of novel methodologies has been motivated by the constraints of conventional regression, such as restrictions on the quantity of predictors and dependence on specific assumptions (LeCroy et al., 2021; Wiemken and Kelley, 2020; Zhang et al., 2009). Recent research has progressively adopted machine-learning (ML) techniques, realizing the need for more advanced analysis and providing the adaptability to record intricate, non-linear interactions (Cheng et al., 2022). The field of machine-learning is becoming increasingly valuable in the context of preventive care. It is praised for its capacity to describe, adapt, learn, predict, and analyze clinical data (Beam and Kohane, 2018). Advances in Artificial Neural Networks (ANN) and Deep Learning (DP) present a way to predict health outcomes more accurately. The main aim of this paper is to carry out a thorough analysis of individuals for obesity, with a specific emphasis on attaining greater precision in predicting levels of obesity risk. Once the relevant datasets have been collected, they undergo thorough preprocessing and feature engineering to refine and prepare the data. Subsequently, seven distinct supervised ML algorithms, namely Logistic Regression, Random Forest (RF), Support Vector Machine (SVM), Light Gradient Boosting Method (LGBM), Extreme Gradient Boosting (XGBoost), Categorical Boosting (CATBoost), and Multi-Layer Perceptron (MLP), were utilized to evaluate essential metrics such as accuracy, precision, recall, and F1-score. We have introduced a new hybrid model called ANN-PSO (Artificial Neural Networks-Particle Swarm Optimization) alongside the traditional ML algorithms. This novel approach combines the optimization techniques of Particle Swarm Optimization (PSO) with the learning abilities of ANN. The incorporation of the ANN-PSO model enhances the complexity of our analysis, leveraging the synergistic effects between optimization techniques and neural network-based learning. The superior performance of this hybrid model compared to traditional algorithms highlights its potential to improve accuracy and effectiveness in predicting obesity. The incorporation of the suggested model brings about a substantial improvement to our approach, providing a promising opportunity for further investigation in the domain of predictive analytics for obesity.

## 2 Literature review

Various studies have explored the application of ML models to predict obesity across different populations and age groups. Diverse methodologies and datasets have been employed, ranging from Electronic Health Records (EHR) and clinical decision support systems to publicly available health data. Researchers have experimented with an array of ML algorithms, including ensemble methods, decision trees, Bayesian models, and SVM. Muhamad Adnan et al. (2012) devised a hybrid methodology that integrates Naïve Bayes (NB) with genetic algorithms to enhance prediction accuracy and optimize parameters. The genetic algorithm optimization resulted in the highest level of accuracy. Significantly, they identified a vulnerability in the NB algorithm pertaining to "zero value parameters." Their preliminary tests showcased the efficacy of their method, correctly identifying 92% of the samples with zero value parameters. Dugan et al. (2015) explored the prediction of early childhood obesity using data from the pediatric clinical decision support system, CHICA. They utilized six ML techniques: Random Tree, RF, J48, ID3, NB, and Bayes Net. Among these, the ID3 algorithm emerged as the most effective, achieving an accuracy of 85% and a sensitivity of 89% after thorough training and evaluation. Montañez et al. (2017) applied ML methods to forecast obesity using publicly accessible genetic profiles. They tested algorithms like SVM, decision tree, decision rule, and k-NN to predict chronic hepatitis susceptibility using Single Nucleotide Polymorphisms (SNPs) data. SVM performed the best, achieving a notable AUC of 90.5%. Zheng and Ruggiero (2017) created a high school student obesity prediction model utilizing nine health-related behaviors. They applied binary LR, an improved decision tree (IDT), weighted K-NN, and ANN model. The results showed that the IDT reached 80.23% accuracy, the ANN 84.22%, and the k-NN 88.92%. Jindal et al. (2018) performed a study that aimed to predict obesity by employing ensemble machine-learning techniques. Their strategy achieved an impressive accuracy of 89.68% in predicting obesity, demonstrating its effectiveness. Hammond et al. (2019) used EHR data and publicly available datasets to predict childhood obesity with models like LR, RF, and GBoost. Their study focused on predicting obesity by age five and reported accuracies of 82% for girls and 76% for boys, with LASSO regression showing particularly strong performance. Singh and Tawfik (2019) utilized data from the UK's Millennium Cohort Study to develop a ML model predicting the likelihood of adolescents developing overweight or obesity. Using BMI values from ages 3, 5, 7, and 11, they achieved a prediction accuracy exceeding 90% for the target class despite dataset imbalance. Taghiyev et al. (2020) introduced a hybrid model combining DT and LR techniques to identify and predict obesity. Their approach involved two stages: feature selection followed by classification. The study found that obesity risk in women rises with age, number of pregnancies, blood pressure, body weight, and blood glucose levels. This model achieved an accuracy of 91.4%. Fu et al. (2020) devised a machine-learning framework for forecasting childhood obesity. They employed health examination data, lifestyle and dietary habits, and anthropometric measurements in their analysis. Out of the 25 features analyzed, the course of BMI *Z*-score during the initial year of a child's life and the mother's BMI at the moment of enrolling in the program were found to be important predictors of childhood obesity. Cervantes and Palacio (2020) utilized SVM, DT, and k-means techniques to categorize obesity levels in people aged 18 to 25. Their goal was to optimize interventions designed to promote a more healthful lifestyle. Thamrin et al. (2021) examined the data from the Indonesian Basic Health Research and utilized ML techniques, including NB, LR, and CART (Classification and Regression Trees). The objective was to determine the risk factors for obesity, and it was discovered that LR model yielded the most accurate results. However, the agreement between the predicted and measured obesity rates was only moderate. Cheng et al. (2021) conducted experiments with eleven classification algorithms, such as LR, MLP, NB, and fuzzy classifier. They achieved a maximum overall accuracy of 70% by employing a random subspace algorithm. Marcos-Pasero et al. (2021) used RF and GBoost algorithms to forecast BMI, leveraging 190 variables from a sample of 221 children aged 6–9 years. These techniques effectively capture complex relationships in high-dimensional data, with RF improving prediction accuracy by averaging multiple decision trees and assessing variable importance via out-of-bag (OOB) error. Zare et al. (2021) predicted obesity in fourth graders using BMI data from kindergarten along with demographic and socioeconomic factors. Their LR and ANN models achieved an accuracy of about 87%, with the kindergarten BMI Z-score emerging as a crucial predictor of future obesity, highlighting the importance of early BMI data. Pang et al. (2021) carried out a study on the prediction of childhood obesity using EHR data. They employed seven different techniques, including XGBoost, which achieved an accuracy of 66.14%. Their study highlighted the significance of using authentic EHR data to support research on interventions for childhood obesity. Rodríguez et al. (2021) proposed an ML model to predict obesity and overweight, using 16 features related to physical condition and diet. Among several algorithms tested, including DT, SVM, k-NN, Gaussian naive Bayes, MLP, RF, gradient boosting, and GXBoost, RF showed the best performance with 78% accuracy. Cheng et al. (2022) used long short-term memory (LSTM) models to predict BMI in children aged 0–4 using EHR data from 2 to 8 visits. It found that five visits were sufficient for accurate predictions, with a combined model achieving an MAE of 0.98 and R2 of 0.72. The final model identified 24 key variables, improving prediction reliability before age 4. A summary of previous works on obesity classification is presented in Table 1.

**Table 1:** Background works summary.

**References**	**Machine-learning methods**	**Dataset**	**Features**	**Accuracy**
Muhamad Adnan et al. (2012)	Naïve Bayes with genetic algorithms	180	19	0.92
Dugan et al. (2015)	Random Tree, Random Forest, J48, ID3, Naive Bayes (NB),	7519	167	0.85
Montañez et al. (2017)	SVM, decision tree, decision rule, MLP, and k-NN	6622	13	0.905
Zheng and Ruggiero (2017)	K-NN	5227	9	0.8882
Jindal et al. (2018)	RF Ensemble method	600	5	0.8968
Hammond et al. (2019)	LR, RF, and GBoost	3449	150	0.82
Singh and Tawfik (2019)	MLP	11110	4	0.91
Taghiyev et al. (2020)	DT + LR	500	26	0.914
Fu et al. (2020)	LightGBM	8269	25	0.74
Cervantes and Palacio (2020)	DT + k-means	178	17	0.98
Thamrin et al. (2021)	Naïve Bayes, LR, and CART	618,89	21	0.72
Cheng et al. (2021)	LR, MLP, naïve Bayes, and fuzzy classifier	7162	5	0.7
Marcos-Pasero et al. (2021)	RF and GBoost	221	190	0.55
Zare et al. (2021)	LR, RF, NN	2147	18	0.87
Pang et al. (2021)	XGBoost	27203	54	0.66
Rodríguez et al. (2021)	RF	211	16	0.78
Cheng et al. (2022)	LSTM	269	24	0.72

While previous works on obesity prediction have strengths like the use of diverse algorithms and large datasets, they often suffer from low accuracy, overfitting, and a lack of adaptability to new data. This study tries to address these weaknesses by applying the ANN-PSO model, which optimizes neural network hyperparameters through Particle Swarm Optimization. This approach enhances both accuracy and robustness, effectively filling the gaps left by traditional machine-learning models in obesity prediction.

## 3 Materials and methods

The primary objective of this study is to employ machine-learning methods to assess an individual's degree of obesity by examining their dietary patterns and physical state. The study categorizes individuals into seven distinct categories based on their BMI, given by Equation 1, which is a key determinant. These categories include underweight, normal weight, overweight level I, overweight level II, obese type I, obese type II, and obese type III.


(1) $$\begin{array}{l} \text{BMI}=\frac{\text{weight (kg)}}{{\text{height (m)}}^{2}} \end{array}$$

This classification confirms the criteria set forth by the WHO guidelines, as delineated in Table 2. The primary objective is to create a prognostic model that precisely classifies individuals into the designated obesity categories. This model has the capacity to make a significant contribution to the early detection and prevention of obesity, establishing the basis for personalized treatment plans that are specifically designed to meet the individual needs of each patient. To ensure a robust evaluation of the predictive models, the dataset was strategically split, with 70% allocated for training purposes, 15% for validation, and the remaining data reserved for testing. The training dataset was subsequently partitioned into ten equivalent subsets for the purpose of 10-fold cross-validation, further enhancing the reliability of the model training and providing a comprehensive assessment of their performance on unseen data. The study employed a diverse set of ML algorithms, including LR, SVM, RF, MLP, LGBM, XGBoost, and CATBoost. The inclusion of this varied array of algorithms enables a comprehensive exploration of the dataset, capturing diverse patterns and nuances. Each algorithm contributes unique strengths, collectively enhancing the study's capacity to predict and classify obesity levels based on dietary habits and physical conditions. In addition to leveraging traditional ML algorithms, the study incorporated a hybrid algorithm known as ANN-PSO. This innovative approach combines Particle Swarm Optimization with Artificial Neural Networks to enhance the optimization process. Following the deployment of ANN-PSO, the study conducted a meticulous comparison, pitting the performance of the hybrid metaheuristic algorithm against various ML models. The primary metric for this comparative analysis was accuracy, providing a comprehensive evaluation of the predictive capabilities of both ANN-PSO and the seven ML models. This approach underscores a holistic exploration, considering both traditional ML methods and advanced hybrid metaheuristic solutions for predicting obesity based on dietary habits and physical conditions.

**Table 2:** BMI classification based on the WHO.

**Class**	**BMI range**
Underweight	Less than 18.5
Normal	18.5–24.9
Overweight	25.0–29.9
Obesity I	30.0–34.9
Obesity II	35.0–39.9
Obesity III	Higher than 40

### 3.1 Overview of datasets

**Figure 1:**
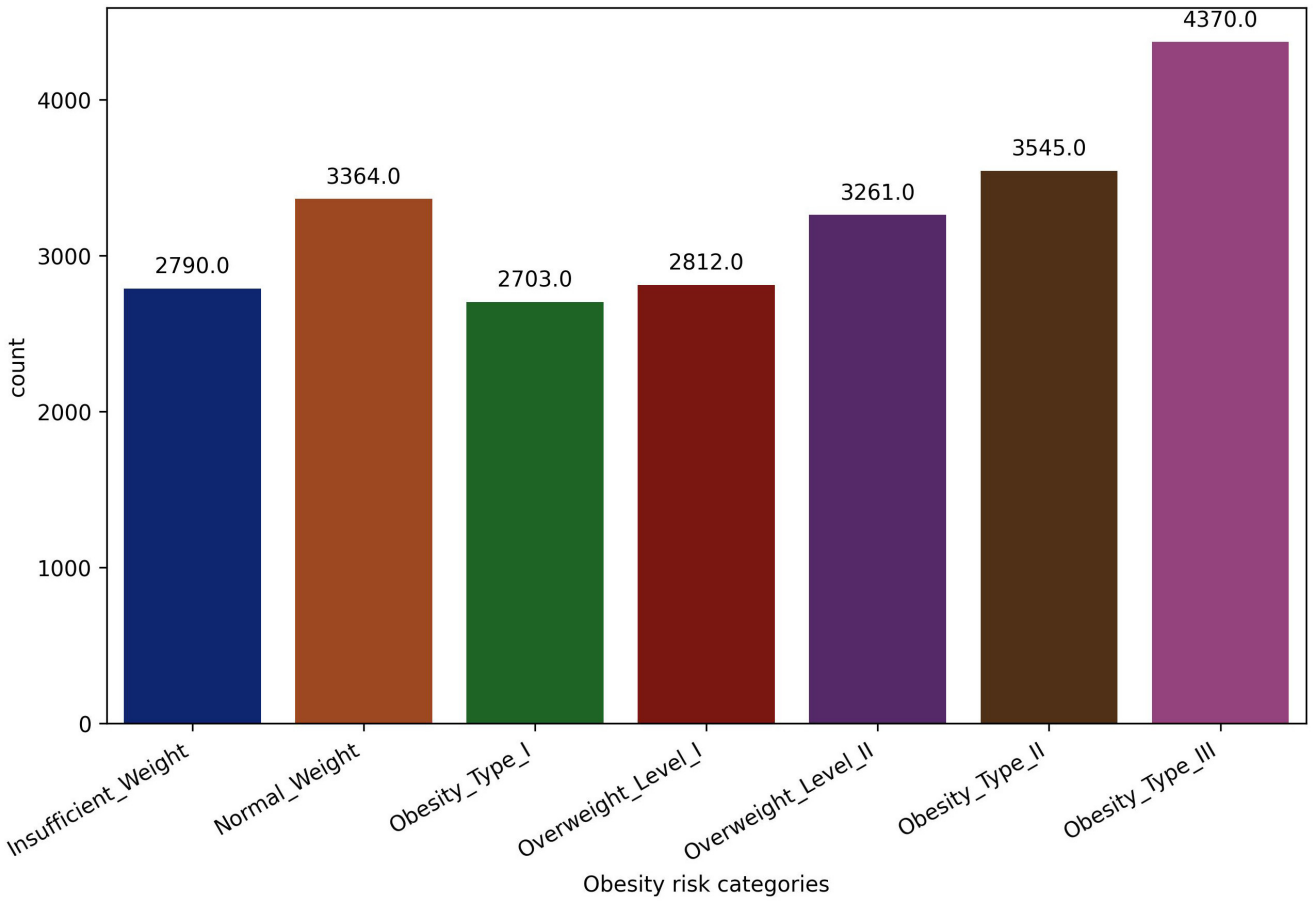
Dataset class distribution.

The dataset contains estimated obesity rates for people from Colombia, Peru, and Mexico who are between the ages of 14 and 61. These people's physical and dietary characteristics vary widely. Data collection was carried out using an online survey platform, where participants who chose to remain anonymous answered a range of questions. Through this process, a total of 17 attributes were obtained for analysis. In the data integration phase of this research project, two datasets, one with 2,111 records and the other with 20,758 records, were seamlessly merged, each possessing identical features. The decision to combine datasets with matching features was deliberate, aiming to create a consolidated dataset that maximizes the richness of the information available. Both datasets shared common variables, facilitating a straightforward merging process (Palechor and Manotas, 2019; Reade and Chow, 2024), offering a holistic perspective and contributing to the robustness and generalizability of the study's findings. The consolidated dataset serves as a comprehensive repository for evaluating obesity levels in individuals, considering both dietary habits and physical conditions. It includes a mix of numeric, binary, and categorical variables. The dataset's focal point is a crucial target variable, providing detailed classifications into distinct obesity levels, ranging from Underweight to various Obesity Types (I, II, and III). Figure 1 presents the distribution of participants across seven distinct obesity risk categories, illustrating the frequencies of each category as determined by the dependent variable. This diverse dataset enables a thorough exploration of the complex interplay between different factors and the nuanced categorization of obesity levels. Table 3 offers a detailed overview of the data variables, including their names, types, and precise definitions. The dataset includes eating habit attributes like high-calorie food and vegetable consumption, number of daily main meals, food consumption between meals, daily water intake, and alcohol consumption. It also covers aspects of physical condition like tracking calories, how often people exercise, how long they spend on technology, and how they get around.

**Table 3:** Dataset overview.

**Features**	**Values**
Gender	Male = 0, Female = 1
Age	Numeric
Height	Numeric
Weight	Numeric
Family with overweight	No = 0/Yes = 1
FAVC (Frequent consumption of high caloric food)	0 = Yes/1 = No
FCVC (Frequent consumption of vegetables)	1,2 or 3
NCP (Number of main meals)	1, 2, 3 or 4
CAEC (Consumption of food between meals)	No = 1, Sometimes = 2, Frequently = 3 or Always = 4
Smoke	No = 0/Yes = 1
CH2O (Consumption of water daily)	1, 2 or 3
SCC (Calories consumption monitoring)	No = 0/Yes = 1
FAF (Physical activity frequency)	0, 1, 2 or 3
TUE (Time using technology devices)	0, 1 or 2
CALC (Consumption of alcohol)	No = 1, Sometimes = 2, Frequently = 3 or Always = 4
MTRANS (Transportation used)	Automobile, motorbike, and public transportation and walking
Obesity level	Insufficient Weight = 1, Normal Weight = 2, Overweight Level I = 3, Overweight Level II = 4, Obesity Type I = 5, Obesity Type II = 6, Obesity Type III = 7

#### 3.1.1 Feature engineering

**Figure 2:**
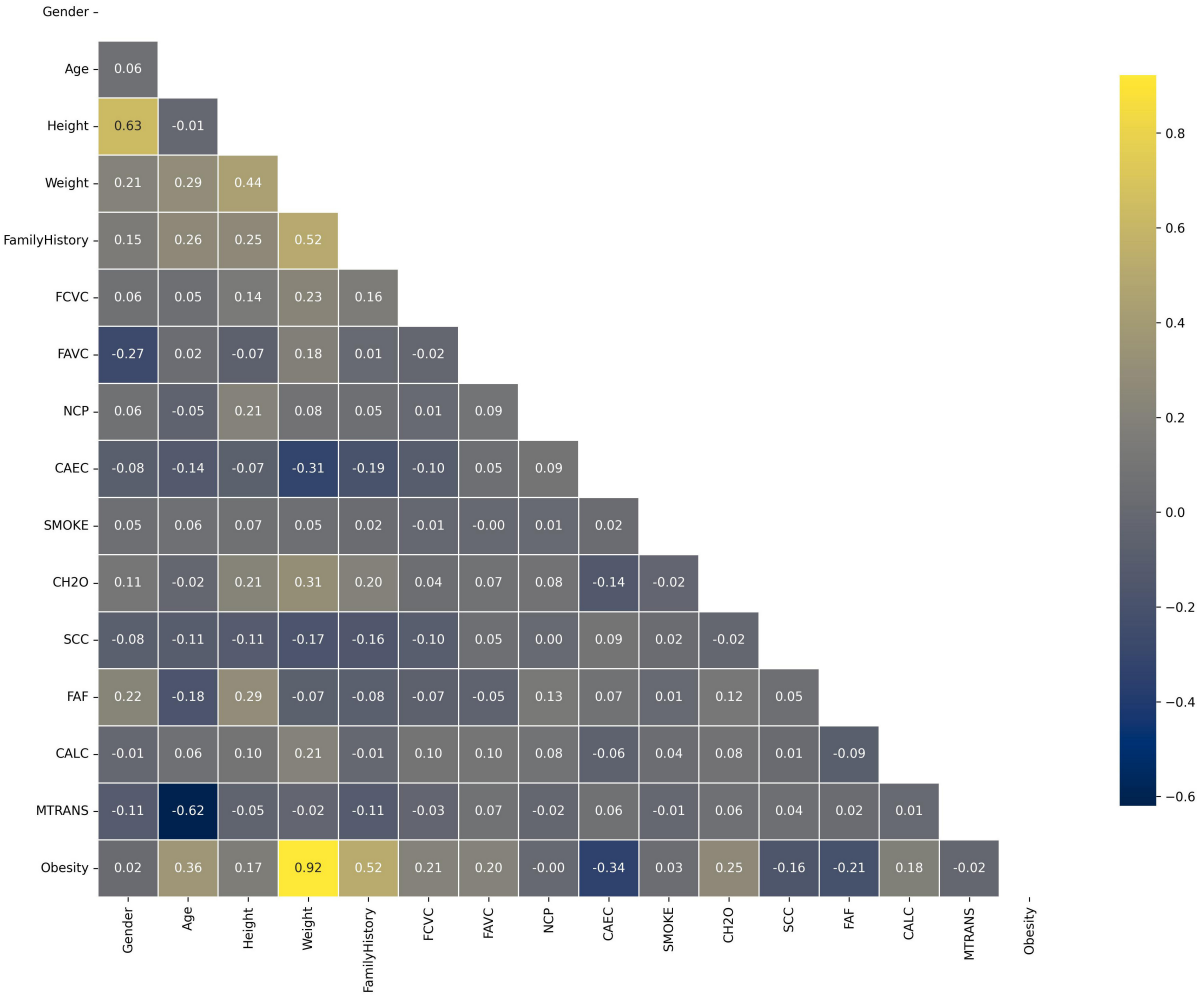
Dataset correlation matrix.

The correlation matrix of the dataset, as it is shown in Figure 2, reveals significant relationships between various features and obesity levels. Notably, weight (0.92) and height (0.15) show positive correlations with obesity, underscoring their direct impact on BMI, a critical indicator of obesity. Family history (0.52) and age (0.35) also exhibit moderate correlations, reflecting the genetic and age-related factors influencing obesity. Negative correlations are observed with CAEC (–0.36), suggesting that certain dietary habits inversely affect obesity levels. In the process of feature engineering and data preprocessing for this study, several meticulous steps were undertaken to enhance the quality and relevance of the dataset for obesity prediction. Initial steps involved the removal of null values, outliers, and duplicates, ensuring data integrity. Subsequently, to enhance the robustness of our analysis and avoid trivial dependencies, we removed height from the dataset. This decision was based on the observation that height, along with weight, can directly influence the calculation of BMI, which in turn affects the dependent variable. Given that BMI is a simplistic measure and can misclassify individuals due to factors such as muscle mass, fat distribution, or metabolic health, our goal was to develop a model that does not rely on BMI for prediction. By excluding height, we aimed to focus on other features within the dataset—such as dietary habits, physical activity, genetic markers, and demographic factors—that provide a more comprehensive understanding of obesity. This approach ensures that our model's predictions are not biased by variables that could trivially affect the outcomes and allows us to explore patterns beyond the direct influence of BMI. Further enriching the dataset, a new feature, "Meal Habits," was created by combining FCVC and NCP, capturing the joint influence of these variables on overall meal habits.


(2) $$\begin{array}{l} \text{Meal Habits}=\text{FCVC}\times \text{NCP} \end{array}$$

Additionally, the interplay between physical activity and sedentary behavior was addressed by introducing the "Activity Balance" feature, combining FAF and TUE. This feature reflects the balance between engagement in physical activity and time spent on technology, providing insights into participants' lifestyles.


(3) $$\begin{array}{l} \text{Activity Balance}=\text{FAF}-\text{ScaleTUE} \end{array}$$

Where Scale (TUE) represents a scaled version of the 'TUE' variable, ensuring compatibility with the FAF values. Also, recognizing the influence of age on technology usage, a "Tech Usage Score" was derived by weighting TUE based on age. This score quantifies the average amount of time a person spends using technology per unit of their age, providing a normalized measure for further analysis.


(4) $$\begin{array}{l} \text{Tech Usage Score}=\frac{\text{ScaleTUE}}{\text{age}} \end{array}$$

The transportation mode feature (MTRANS) was thoughtfully modified to categorize modes based on physical activity, aligning with the research focus on obesity prediction. Lastly, recognizing the imbalance within the obesity level as a target feature, as shown in Figure 1, the Synthetic Minority Over-sampling Technique (SMOTE) was employed to intelligently generate synthetic instances of the minority class, mitigate class imbalance, and promote a more representative distribution (Chawla et al., 2002). These comprehensive feature engineering and data preprocessing steps contribute to a refined and balanced dataset, setting the foundation for robust obesity prediction models in this research.

### 3.2 Machine-learning supervised algorithms

#### 3.2.1 Logistic regression

Logistic regression is a widely used supervised machine-learning classifier that estimates the probability of an event occurring based on a dataset of independent variables. For multiclass scenarios, multinomial logistic regression is employed. The algorithm works by fitting a logistic function to the data to determine the likelihood of different classes. Multinomial logistic regression's ability to handle multiclass problems makes it a valuable tool in various applications requiring classification tasks (LaValley, 2008). Unlike binary LR (Kanade, 2022b; Sperandei, 2014), which uses the sigmoid function, multinomial logistic regression generalizes the logistic function to accommodate multiple classes.

#### 3.2.2 Support Vector Machine

One of the best types of supervised ML algorithms is the Support Vector Machine, which is particularly effective in solving intricate problems related to classification, regression, and outlier detection. It accomplishes this by establishing optimal decision boundaries among data points according to their labels or outputs. SVMs aim to identify hyperplanes that distinctly segregate data points, maximizing the margin between support vectors to enhance classification accuracy (Kanade, 2022a). By transforming input data into higher-dimensional feature spaces using kernel functions, SVMs can effectively handle both linear and non-linearly separable data. SVMs' effectiveness lies in their ability to find optimal hyperplanes that maximize margins between different classes while minimizing classification errors, making them valuable in diverse applications across industries like healthcare, natural language processing, signal processing, speech, and image recognition fields (Kanade, 2022a; Suthaharan, 2016).

#### 3.2.3 Random Forest

The Random Forest algorithm is a powerful method that enhances the accuracy of classification and regression tasks. It works by creating many decision trees during training. Each tree uses a random selection of features and data and then combines their predictions for more reliable results (Rigatti, 2017; Schonlau and Zou, 2020). The ultimate forecast is established by consolidating the forecasts of individual trees, either by means of voting for classification or averaging for regression. RF mitigates overfitting by introducing randomness in the feature selection process and promoting diversity among the constituent trees. This algorithm is known for its remarkable performance, scalability, and ability to manage large datasets with many dimensions. Its versatility, resilience to noise (Kursa, 2014), and capability to capture complex relationships in data make RF a popular choice in various ML applications across different domains.

#### 3.2.4 XGBoost

XGBoost, short for Extreme Gradient Boosting, is a well-known library that implements distributed gradient boosting decision trees (GBDT). It is widely recognized for its exceptional efficiency, flexibility, and portability (Chen and Guestrin, 2016). GBDT is an ensemble learning algorithm employing decision trees, much like the RF, suitable for both classification and regression tasks. However, the key divergence lies in the methodology employed for building and combining these trees (Ramraj et al., 2016). Operating under a parallel tree-boosting framework, XGBoost excels in swiftly and accurately addressing various data science challenges. One of its standout features lies in the algorithm's ability to apply regularization through both L1 and L2 penalties, crucial for preventing overfitting and enhancing model generalization (Choudhuri, 2022).

#### 3.2.5 LGBM

In traditional implementations of GBDT, the amount of computing effort grows in direct proportion to the number of features and the size of the sample dataset. LightGBM employs a novel technique called "gradient-based one-side sampling", facilitating faster training by focusing on instances with larger gradients (Ke et al., 2017). Additionally, LGBM adopts a histogram-based learning approach, enhancing computational efficiency by discretizing continuous features. These optimizations contribute to LGBM's reputation for being one of the fastest and most efficient gradient-boosting frameworks, making it a favored choice for various ML applications.

#### 3.2.6 CATBoost

Categorical Boosting stands out as a formidable gradient-boosting algorithm meticulously crafted for ML endeavors. Its exceptional strength lies in its adeptness at handling input spaces that incorporate categorical features, making it particularly well-suited for tasks where such features play a crucial role in the learning process (de la Bourdonnaye and Daniel, 2022; Dorogush et al., 2017, 2018). The algorithm implements innovative techniques like ordered boosting, oblivious trees, and a specialized algorithm for dealing with numerical and categorical features simultaneously. One of its notable strengths lies in its efficient handling of categorical features. CATBoost algorithm stands out from others as it eliminates the need for preprocessing or one-hot encoding when dealing with categorical variables. This simplifies the training process and minimizes the chances of data leakage.

#### 3.2.7 Multi-layer perception

The MLP, a commonly used type of ANN in machine-learning, comprises multiple layers: an input layer, one or more hidden layers, and an output layer (Dirik, 2023; Disse et al., 2018). The hidden layers introduce complexity, enabling the network to learn intricate patterns from input data. Each node in these layers, excluding the input layer, utilizes nonlinear activation functions, introducing non-linearity into the model. One of the key strengths of MLP lies in its application of supervised learning. Backpropagation, a technique integral to MLP training, involves continuously fine-tuning weights and biases to reduce the gap between predicted and observed outputs. This iterative learning process allows MLP to effectively capture and represent complex relationships in data, making it a versatile tool in various ML applications.

### 3.3 Hybrid ANN-PSO approach

The use of backpropagation as the primary learning algorithm in ANN models does not always ensure optimal solutions, as it can get stuck in sub-optimal weight configurations, hindering the achievement of favorable outcomes. Challenges in simple ANNs include identifying the suitable network architecture for a specific problem, slow learning processes requiring numerous iterations for convergence, rapid forgetting when encountering infrequently seen examples, and the lack of foundational first principal knowledge (Ungar et al., 1990). Particle Swarm Optimization is a population-based algorithm that is renowned for its distinct proficiency in locating optimized solutions and can play a pivotal role in achieving optimal network topology and weights. Consequently, this paper employs PSO as the training algorithm, aiming to determine a set of weights with minimum cost function value, specifically the mean square error (MSE), and consequently increases the performance of the model.

#### 3.3.1 Particle swarm optimization

PSO operates on the principles of cultural adaptation, drawing inspiration from behaviors observed in bird flocks (Eberhart and Kennedy, 1995). Each particle in the swarm assesses its neighbors, compares itself to others, and emulates superior particles, fostering a collaborative approach. Unlike competitive heuristic algorithms, such as genetic algorithms (Goldberg and Richardson, 1987) or ant colony optimization (Dorigo et al., 2006), PSO aims to converge toward the global optimum of a multidimensional and potentially nonlinear function. In PSO, swarms of particles represent potential solutions, moving through the search space to find the global optimum. Each particle maintains its best position (*P*_best_) and contributes to the swarm's global best position (*G*_best_). The optimization process relies on the collaboration between particles, with their velocities updated based on individual and global best positions, employing both social and cognitive components randomly. The algorithm can initialize particle populations with either random or heuristic positions, terminating after a defined number of iterations (Shi and Eberhart, 1998). During each iteration, each particle adjusts its speed and position based on the following formulas:


(5) $$\begin{array}{l} v_{i}^{k+1}=\omega v_{i}^{k}+c_{1}r_{1}\left(P_{\text{best},i}-x_{i}^{k}\right)+c_{2}r_{2}\left(G_{\text{best}}-x_{i}^{k}\right) \end{array}$$

(6) $$\begin{array}{l} x_{i}^{k+1}=x_{i}^{k}+v_{i}^{k+1} \end{array}$$

The position of each particle is expressed as a vector in a multidimensional space, where each dimension of the vector corresponds to a specific coordinate *x*_*i*_. Likewise, the speed of every particle, represented as *v*, is described as a vector with *x*_*i*_ components. The variable *k* represents the number of iterations performed during the optimization process. The constants *c*_1_ and *c*_2_ determine the rate at which the particles' velocities change, affecting the amount of learning that occurs in each iteration. Furthermore, *r*_1_ and *r*_2_ denote random variables that follow a uniform distribution between 0 and 1. These variables contribute to the stochastic nature of the optimization process and improve its ability to explore different possibilities. The PSO algorithm adjusts the inertia weight using the following formula:


(7) $$\begin{array}{l} \omega^{k}=\omega_{\text{max}}-\left(\frac{\omega_{\text{max}}-\omega_{\text{min}}}{k_{\text{max}}}\right)\times k \end{array}$$

where ω^*k*^ represents the inertia weight used in each iteration, *k*_max_ represents the maximum number of iterations, and ω_max_ and ω_min_ represent the maximum and minimum values of the inertia weight, respectively.

**Figure 3:**
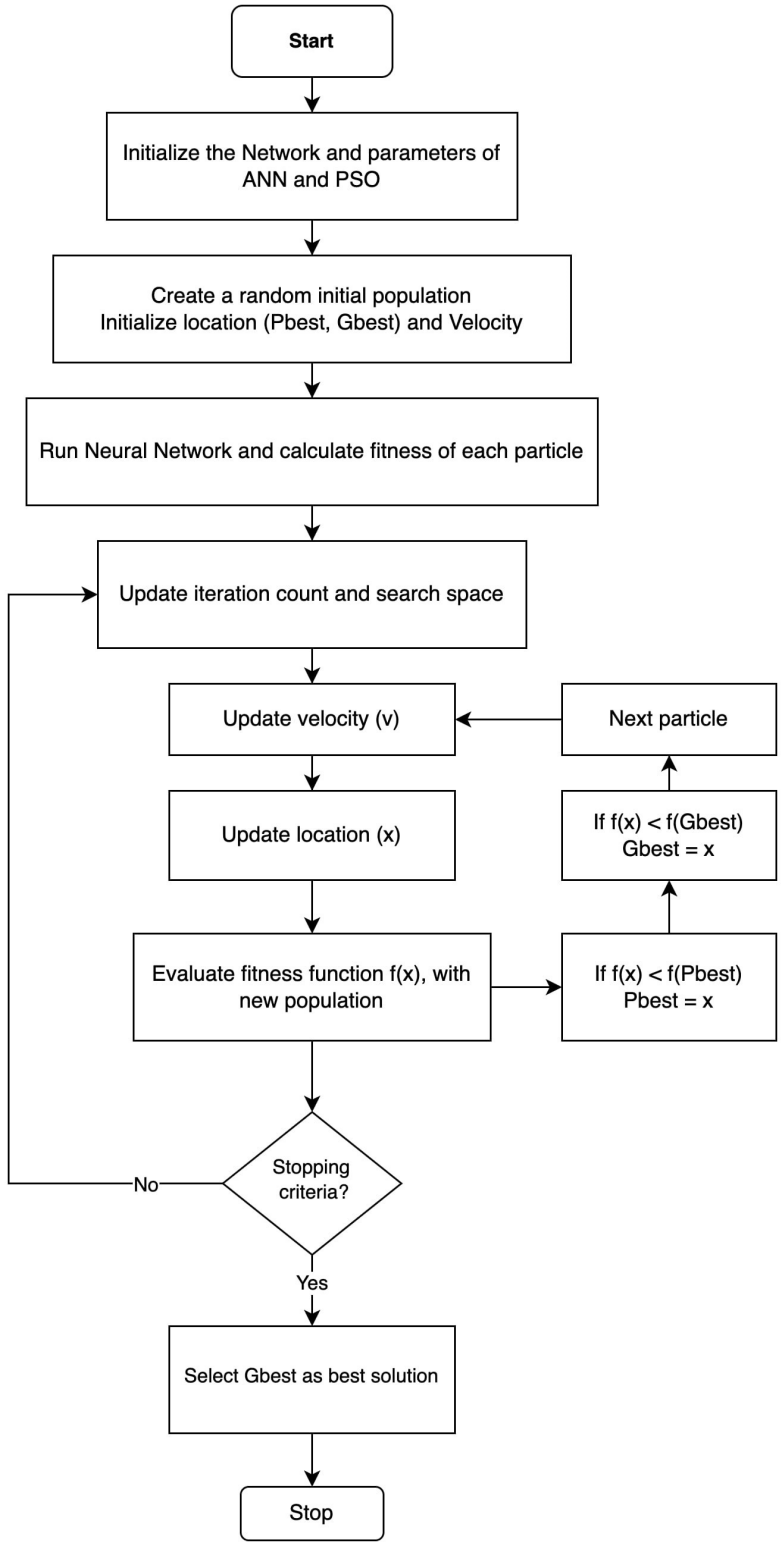
ANN-PSO model flowchart.

The hybrid ANN-PSO model combines the strengths of ANN, which are powerful for learning complex patterns from data, with PSO, known for efficiently finding optimal solutions in large search spaces. This combination synergistically enhances the model's ability to handle complex optimization tasks. This powerful ML technique begins by initializing a neural network with random weights. It then utilizes the PSO algorithm to determine the optimal set of weights for the neural network. The ANN is responsible for performing learning and processing tasks, while the PSO algorithm is employed to discover the optimal weights that improve the performance of the neural network. The integration of this optimization algorithm in the ANN-PSO model enhances the speed at which superior solutions are found, surpassing the reliance solely on ANN learning. This integrated model combines the individual strengths of both algorithms, resulting in enhanced optimization and training results. Figure 3 illustrates the schematic representation of the proposed hybrid model.

### 3.4 Hyper parameter tuning

In the pursuit of optimizing predictive performance, hyperparameter tuning played a pivotal role in this research, which involved the exploration of all ML algorithms applied in this research. Hyperparameter tuning is a critical step in fine-tuning the configuration of these models to achieve optimal results. By systematically adjusting parameters such as learning rates, regularization terms, and tree depths, we aimed to identify the most effective combinations for each algorithm. The process involved a comprehensive search across the hyperparameter space, utilizing techniques like grid search or randomized search, to enhance the models' predictive accuracy and generalization across diverse datasets. The considered hyperparameter values for each model, which were explored during the grid and random search processes, are detailed in Table 4. This table provides an overview of the range of hyperparameters tested, offering insight into the tuning process that led to the selection of the optimal parameters presented in Table 5.

**Table 4:** Hyperparameter values for grid and random search.

**Model**	**Hyperparameter**	**Considered values**
Logistic regression	Penalty	l1, l2
	C	[1, 10]
	Max Iterations	[100, 1000]
SVM	C	1–10
	Kernel	Linear, poly, RBF (radial basis function), sigmoid
	Gamma	Scale, auto
LGBM	Random State	42, 101, 202
	Learning Rate	[0.001, 0.1]
	n_estimators	[100, 1000]
	lambda_l1	[0.001, 0.05]
	lambda_l2	[0.001, 0.05]
	max_depth	[1, 20]
	colsample_bytree	[0.1, 1]
	subsample	[0.1, 1]
XGBoost	Learning Rate	[0.001, 1]
	n_estimators	[100, 1000]
	max_depth	[1, 20]
	min_child_weight	[10, 30]
	gamma	[1e-8, 1]
	colsample_bytree	[0, 1]
	subsample	[0, 1]
	reg_alpha	[1e-8, 10]
	reg_lambda	[1e-8, 10]
CATBoost	Learning Rate	[0.001, 0.1]
	Depth	[1, 20]
	l2_leaf_reg	[0.001, 1]
	Bootstrap Type	Bayesian, Bernoulli, Poisson
	Random Strength	[1e-8, 1e-4]
	n_estimators	[100, 1000]
MLP	Hidden Layers	[3, 5]
	Dense Layer Neurons	32, 64, 128, 256, 512
	Activation Function	ReLU, Sigmoid, Tanh
	Kernel Initializer	HeNormal, GlorotUniform
	Optimizer	Adam, SGD, RMSProp
	Learning Rate	[0.0001, 0.1]
PSO	Wmax	[0.6, 0.9]
	Wmin	[0.1, 0.5]
	Cognitive Coefficient	1, 1.5, 2

**Table 5:** Best parameters for different algorithms.

**Algorithm**	**Best parameters**
LR	Penalty = l2; C = 1; Max iteration = 200
SVM	C = 1; Kernel = radial basis function; gamma = scale
LGBM	random_state = 42; learning_rate = 0.01197; n_estimators = 509; lambda_l1 = 0.00972; lambda_l2 = 0.03854; max_depth = 11; colsample_bytree = 0.73643; subsample = 0.95299
XGBoost	learning_rate = 0.09703; n_estimators = 392; max_depth = 10; min_child_weight = 23.04133; gamma = 0.00038; colsample_bytree = 0.36017; subsample = 0.64307; reg_alpha = 0.03715; reg_lambda = 3.3051e-08
CATBoost	learning_rate = 0.07739; depth = 5; l2_leaf_reg = 0.100297; bootstrap_type = Bayesian; random_strength = 3.2419e-08; n_estimators = 501
MLP	Hidden_layer = (128-64-32); Activation_Function = ReLu; Kernel_Initializer = HeNormal; Optimizer = Adam; Learning_Rate = 0.001
PSO	cognitive_coefficient = 2; social_coefficient = 2; maximum_inertia_weight (Wmax) = 0.9; minimum_inertia_weight (Wmin) = 0.2; number_of_particles = 40

## 4 Results and discussion

### 4.1 Performance indicators

The proposed model's effectiveness in classifying obesity was assessed using four metrics: Accuracy, Precision, Recall, and F1 Score. Accuracy is an important measure that quantifies the percentage of correctly predicted samples out of the total. True positives (*tp*) refer to instances where the model accurately predicts positive categories, while false positives (*fp*) refer to instances where the model inaccurately predicts positive categories. On the other hand, true negatives (*tn*) refer to cases where the model correctly predicts negative categories, whereas false negatives (*fn*) occur when the model incorrectly predicts negative categories. Precision is a measure of the model's ability to correctly identify positive cases (*tp*) among all predicted positives in its predictions (*tp* and *fp*). A higher precision value indicates more accurate detection of positive cases and better overall performance. Recall assesses how well the model identifies *tp* instances among all actual positives (*tp* and *fn*). A high recall indicates the model effectively captures a significant proportion of positive cases, minimizing the likelihood of overlooking *fn*. Simultaneously, a significant F1-Score indicates that the model achieves a harmonious equilibrium between precision and recall, suggesting a comprehensive and balanced performance.

### 4.2 Experimental results

Machine-learning models, encompassing LR, SVM, RF, LGBM, XGBoost, CATBoost, and MLP were deployed to predict obesity across seven categories. The accuracy results, outlined in Table 6, demonstrate varied performances among these models. Notably, RF, LGBM, XGBoost, CATBoost, and MLP exhibit high accuracies ranging from 85 to 89%, showcasing substantial predictive capabilities in discerning between different obesity classes. However, the standout performer is the proposed hybrid model, ANN-PSO, achieving an impressive accuracy of 91.79%. The proposed hybrid model surpasses individual algorithms, highlighting its exceptional ability to predict different obesity classes. Beyond accuracy, Table 6 presents a detailed classification report, incorporating overall accuracy, precision, recall, and F-1 score for each model and obesity category.

**Table 6:** Results from different machine-learning models.

**Model**	**Categories**	**Insufficient weight**	**Normal weight**	**Overweight level I**	**Overweight level II**	**Obesity type I**	**Obesity type II**	**Obesity type III**
LR	Precision	0.91	0.72	0.65	0.63	0.78	0.97	1
	Recall	0.84	0.76	0.71	0.62	0.77	0.93	0.99
	F-1 score	0.88	0.74	0.68	0.62	0.78	0.95	1
	Accuracy	0.8072						
SVM	Precision	0.9	0.77	0.72	0.76	0.79	0.97	0.99
	Recall	0.89	0.78	0.75	0.7	0.86	0.94	1
	F-1 score	0.89	0.78	0.74	0.73	0.82	0.96	0.99
	Accuracy	0.844						
RF	Precision	0.94	0.85	0.79	0.085	0.88	0.97	1
	Recall	0.93	0.84	0.83	0.79	0.91	0.97	1
	F-1 score	0.93	0.85	0.81	0.82	0.9	0.97	1
	Accuracy	0.8968						
LGBM	Precision	0.92	0.85	0.81	0.79	0.89	0.97	1
	Recall	0.94	0.83	0.77	0.84	0.89	0.98	1
	F-1 score	0.93	0.84	0.79	0.82	0.89	0.97	1
	Accuracy	0.8916						
XGBoost	Precision	0.93	0.84	0.81	0.8	0.88	0.97	1
	Recall	0.94	0.83	0.78	0.83	0.88	0.98	1
	F-1 score	0.93	0.84	0.8	0.82	0.88	0.97	1
	Accuracy	0.8903						
CATBoost	Precision	0.93	0.84	0.82	0.81	0.9	0.97	1
	Recall	0.94	0.84	0.79	0.85	0.88	0.98	1
	F-1 score	0.93	0.84	0.8	0.83	0.89	0.97	1
	Accuracy	0.8948						
MLP	Precision	0.91	0.79	0.75	0.73	0.88	0.95	1
	Recall	0.88	0.8	0.76	0.76	0.84	0.97	1
	F-1 score	0.9	0.79	0.75	0.75	0.86	0.96	1
	Accuracy	0.858						
ANN-PSO	Precision	0.95	0.88	0.86	0.84	0.92	0.98	1
	Recall	0.94	0.88	0.84	0.87	0.91	0.98	1
	F-1 score	0.95	0.88	0.85	0.86	0.91	0.98	1
	Accuracy	0.9179						

**Figure 4:**
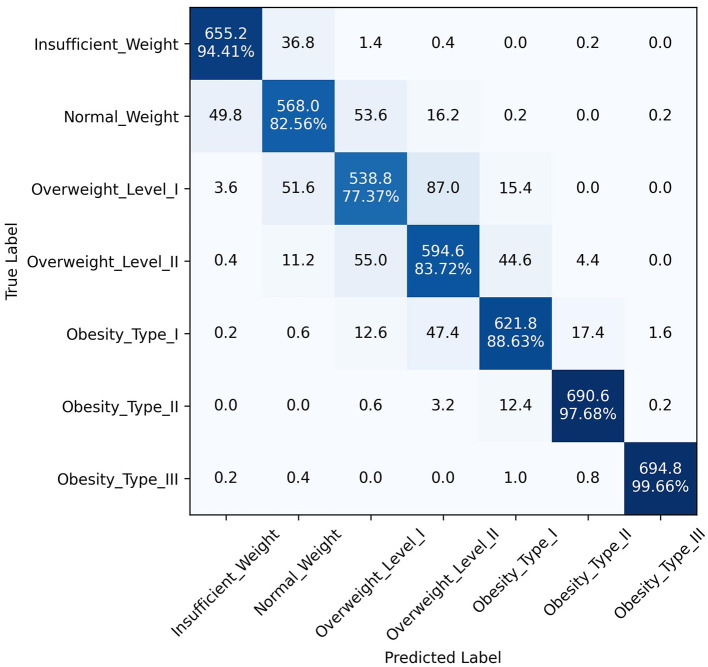
Confusion matrix, LGBM model.

**Figure 5:**
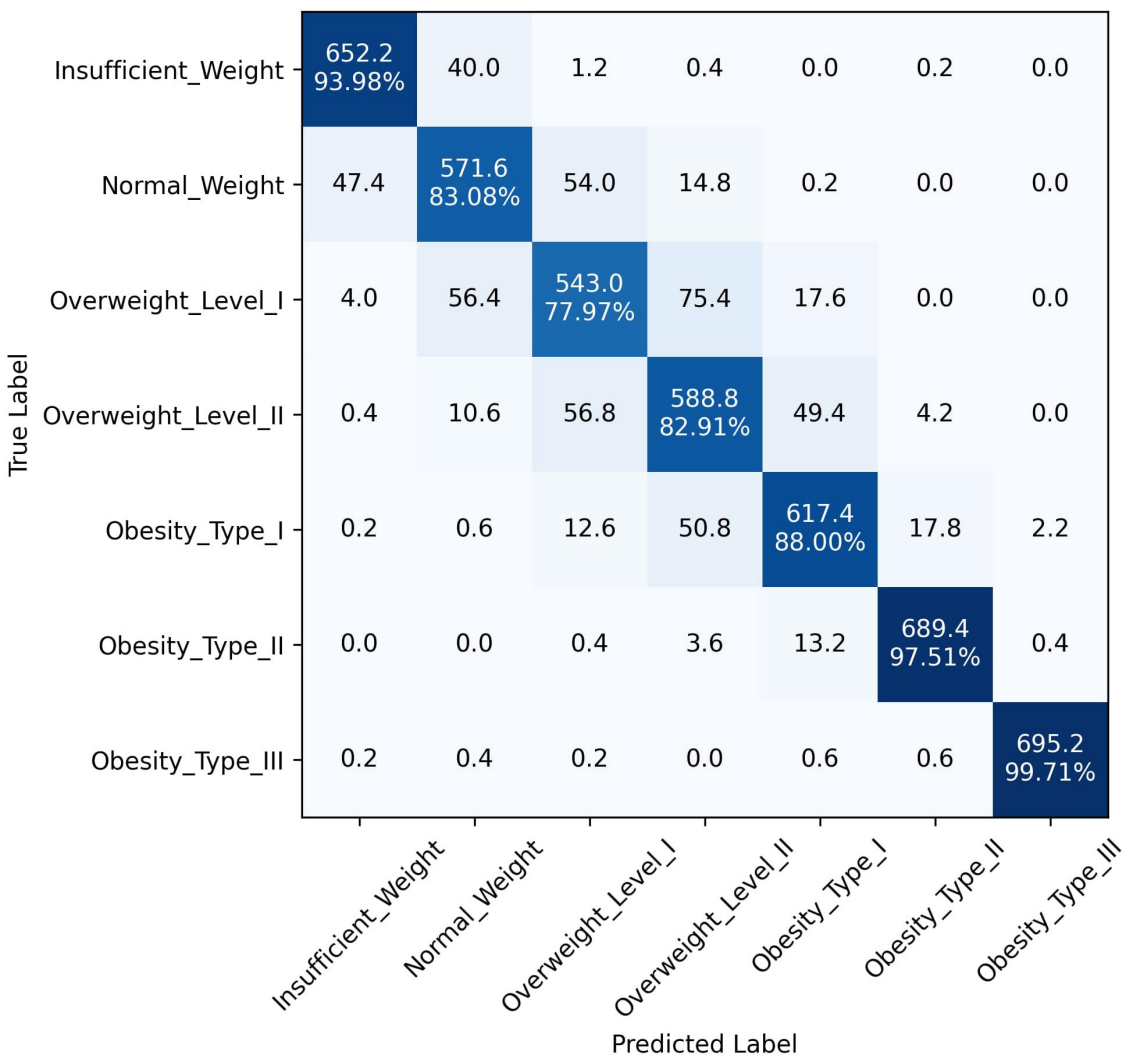
Confusion matrix, XGBoost model.

**Figure 6:**
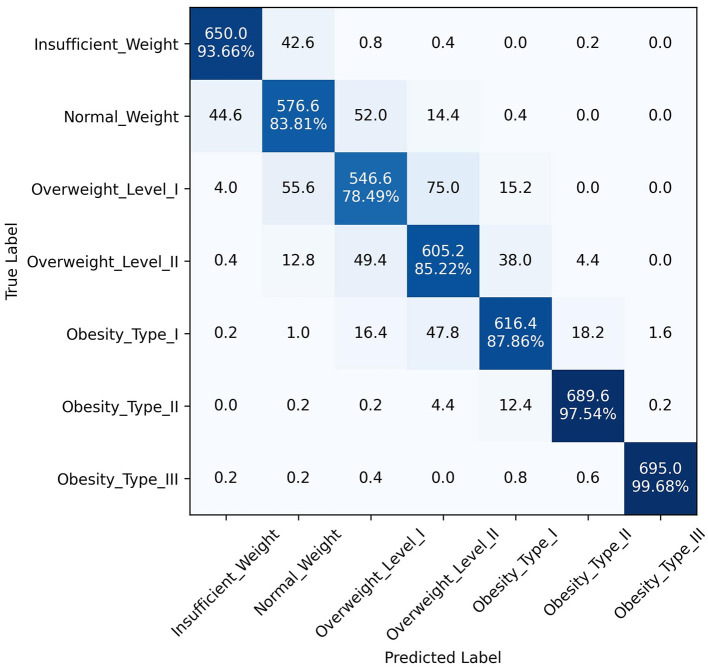
Confusion matrix, CATBoost model.

**Figure 7:**
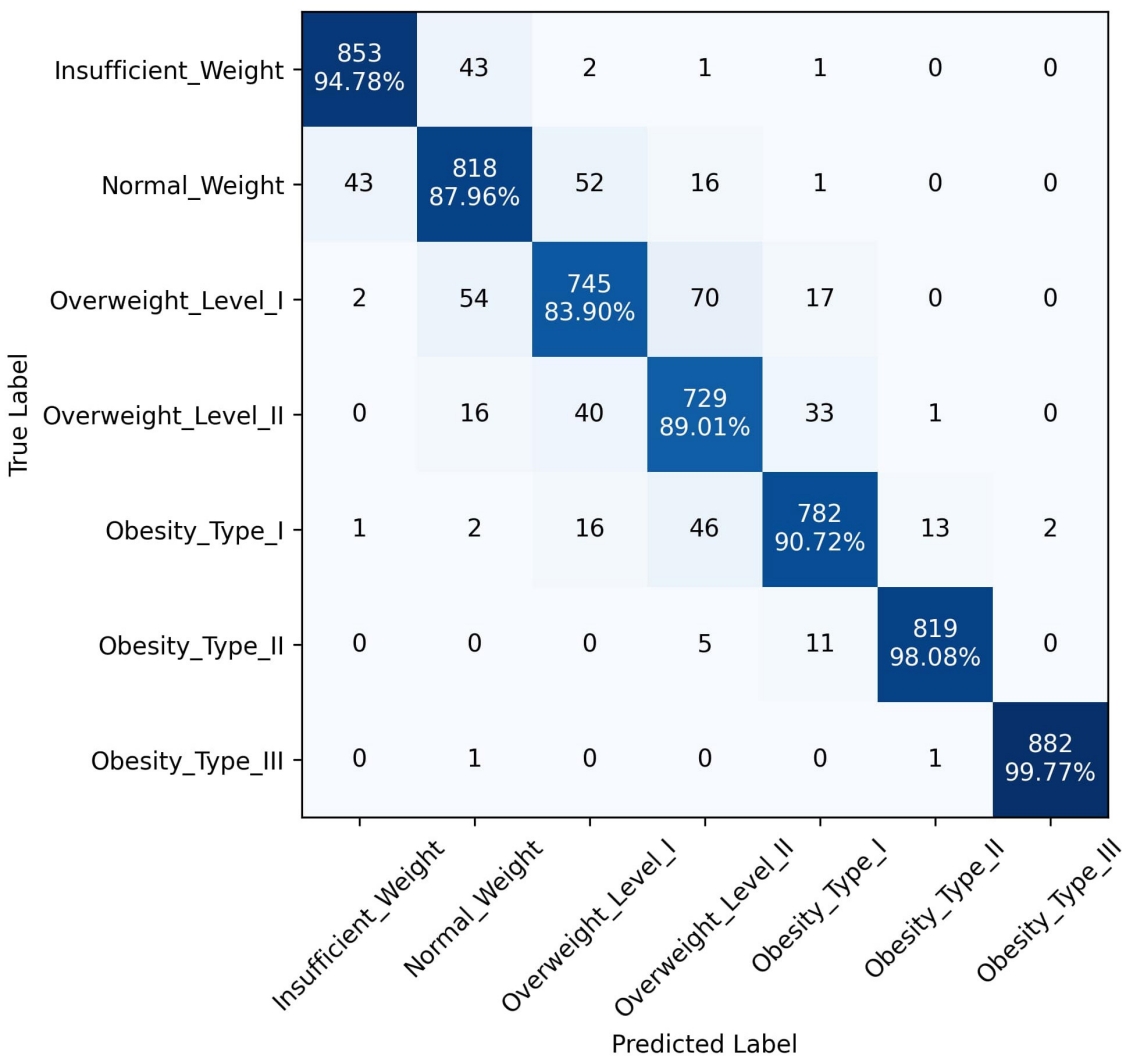
Confusion matrix of ANN-PSO model.

The superior performance of the ANN-PSO model in predicting different levels of obesity across the seven classes can be attributed to the synergistic combination of neural networks and a metaheuristic algorithm like PSO. While ANNs are powerful in learning complex patterns and representations from data, the PSO algorithm further optimized the network weights and architecture, leading to improved generalization and predictive capabilities. A closer examination of the class-wise performance metrics reveals the robustness of the proposed model. It exhibited consistently high precision, recall, and F1 scores across all classes, including the more challenging "Overweight Type I," "Overweight Type II," and "Obesity Type I" categories. This is a significant advantage over the other models, which tended to perform well in the majority classes but struggled with the minority, severe obesity classes. In addition to quantitative metrics, Figures 4–6 depict confusion matrices for the top three models: LGBM, XGBoost, and CATBoost, respectively, providing a visual representation of their classification performance across obesity categories. Figure 7 enhances this analysis by presenting the confusion matrix for the ANN-PSO hybrid model. These matrices offer insights into *tp*, *tn*, *fp*, and *fn*, contributing to a nuanced assessment of model behavior. The inclusion of both quantitative metrics and visual representations ensures a comprehensive evaluation of each model's performance in the context of obesity classification. The ANN-PSO model's confusion matrix stands out with high diagonal values, representing the correctly classified instances for each class. Notably, even for the minority classes like "Overweight level I" and "Overweight level II," the proposed model demonstrates a high *tp* rate, with minimal misclassifications into other classes. In contrast, the confusion matrices for the LGBM, XGBoost, and CATBoost models reveal higher off-diagonal values, particularly for the Overweight classes. This indicates a higher tendency to misclassify instances from these minority classes into other ones, which can have significant implications for obesity risk assessment and treatment planning.

**Figure 8:**
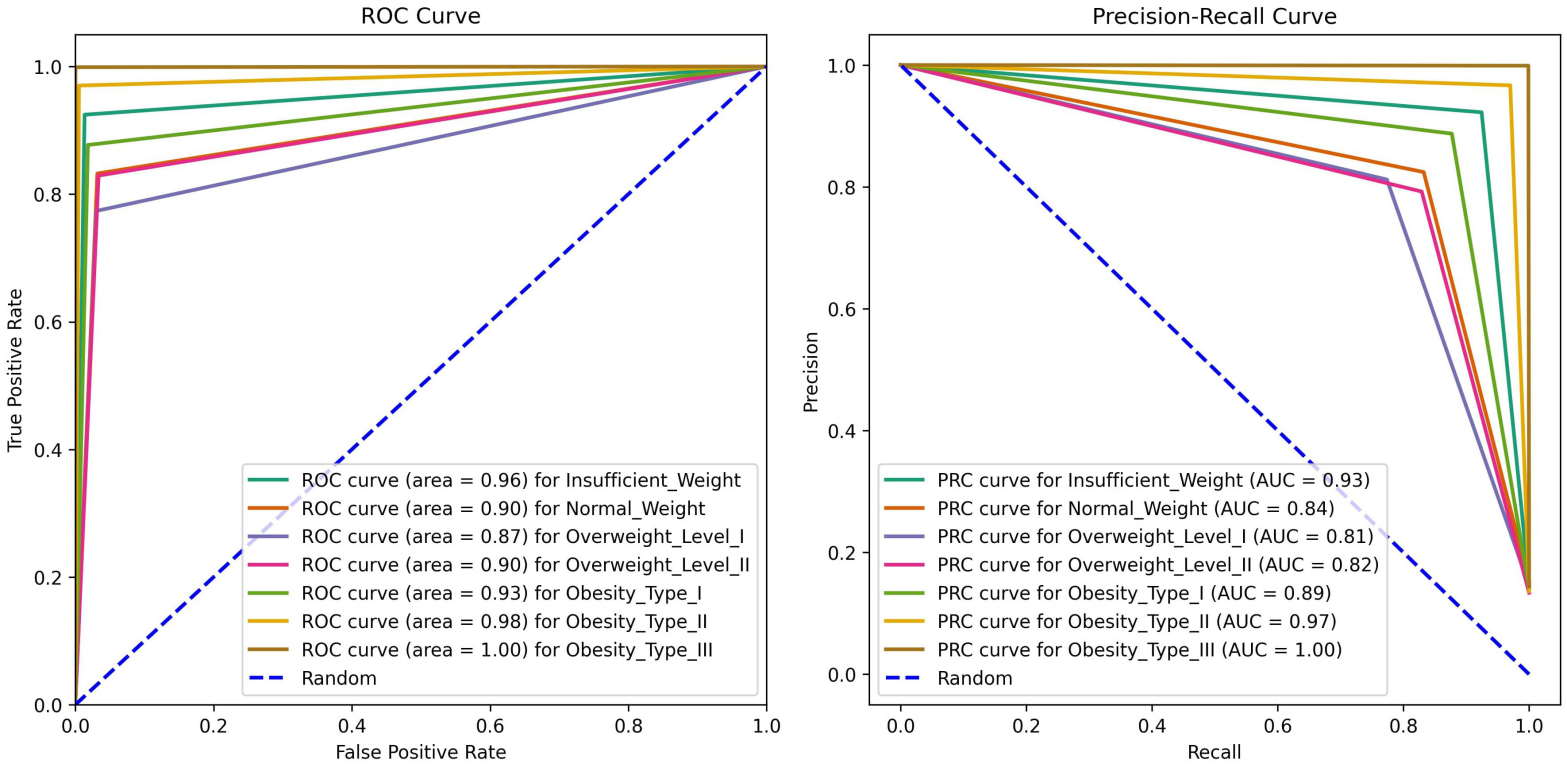
ANN-PSO model, ROC, and Precision-Recall.

In addition to the classification report and confusion matrix, further insights into the performance of the ANN-PSO model are provided through the presentation of ROC (Receiver Operating Characteristic) and Precision-Recall curves in Figure 8. The ROC curve shows how well a classification model distinguishes between positive instances it correctly identifies (*tp* rate) and negative instances it incorrectly identifies as positive (*fp* rate) across various threshold settings. A curve that closely hugs the upper-left corner signifies superior performance, where the model achieves high sensitivity while maintaining low *fp* rates. The overall ability of the model to tell the difference is measured by the area under the curve (AUC) of ROC. A higher AUC indicates better performance in distinguishing between classes, making it a key metric in evaluating the effectiveness of classification models. In the context of the ANN-PSO model, a higher AUC-ROC in Figure 8 suggests robust discrimination power across obesity categories. Simultaneously, the Precision-Recall curve illustrates how a classification model balances its precision against recall across varying thresholds used for classification. A curve that closely follows the upper-right corner indicates high precision and recall. The area under the Precision-Recall curve (AUC-PR) is a valuable metric, emphasizing the model's ability to correctly identify positive instances while minimizing false positives. For the ANN-PSO model, a larger AUC-PR in Figure 8 signifies effective performance in handling imbalanced classes and accurately predicting instances of obesity.

**Figure 9:**
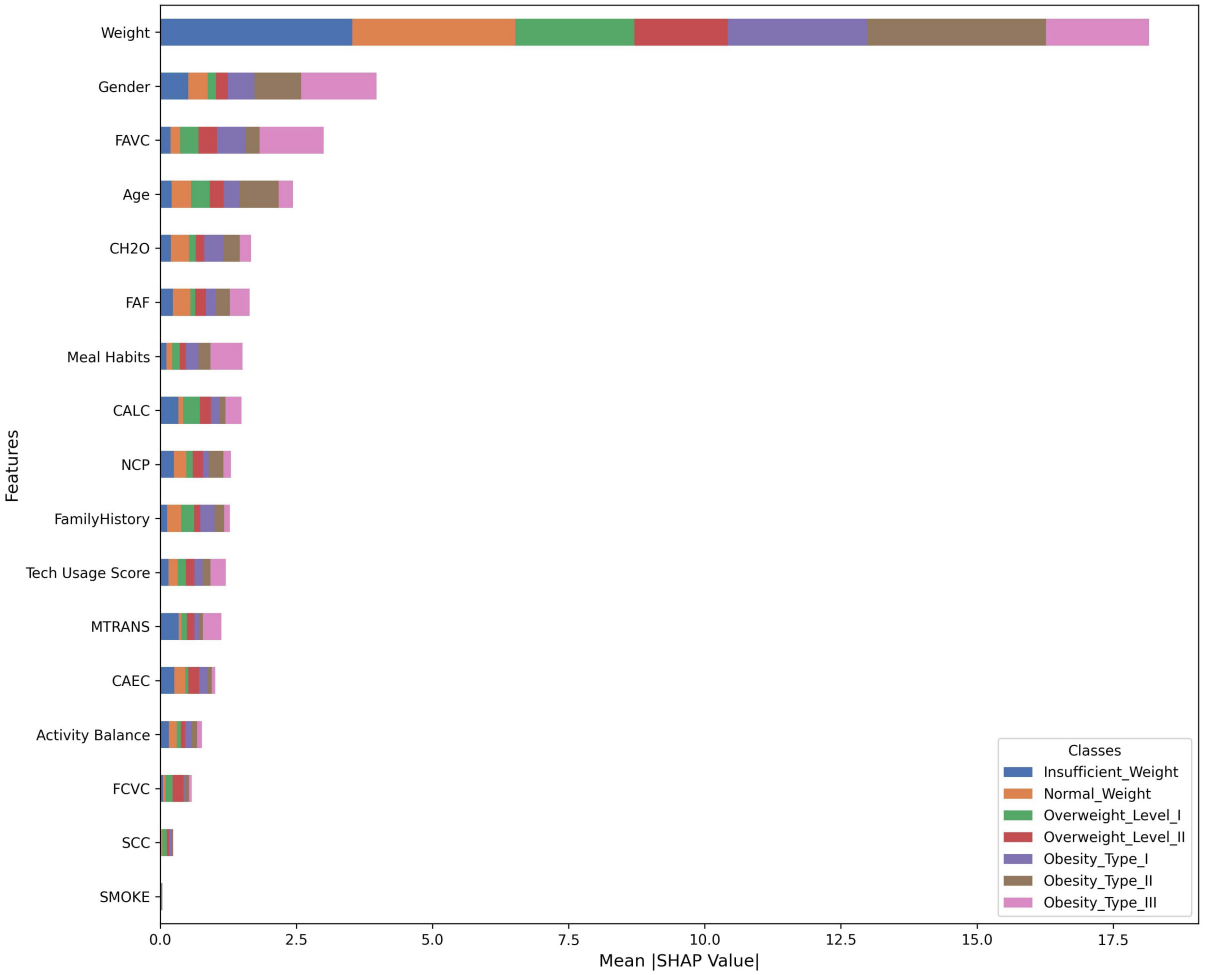
SHAP feature importance for all classes.

To further understand the dynamics of the proposed model in predicting obesity, a feature importance plot, illustrated in Figure 9, was generated using SHAP (SHapley Additive exPlanations). SHAP is a powerful game theory-based approach used to explain the output of ML models (Lundberg and Lee, 2017). SHAP provides a unified measure of feature importance that is both consistent and locally accurate. It helps interpret complex models by assigning each feature an importance value for a particular prediction, indicating how much each feature contributes, positively or negatively, to the model's output. This allows for both global interpretability (overall feature importance) and local interpretability (feature importance for individual predictions). By using SHAP, we are shedding light on the contribution of each input variable to the model's decision-making process. Understanding feature importance not only contributes to model interpretability but also provides actionable insights for decision-makers seeking to comprehend the factors influencing obesity predictions in the ANN-PSO model. It reveals the relevance and impact of each feature on the target parameter (obesity in this case). Features with higher importance play a more significant role in influencing the model's predictions. This information is invaluable for both interpretability and potential feature engineering. While the results achieved by the proposed model are impressive, employing SHAP adds an extra layer of understanding and confidence in the model's predictions. Figure 9 provides several key insights through the feature importance plot. Weight emerges as the dominant feature for classifying obesity levels, showing significant importance across all obesity classes, particularly for higher obesity levels. This result is reasonable and expected, as weight is widely recognized as a crucial parameter in assessing obesity. Gender is the second most important feature, suggesting distinct obesity classification patterns between males and females. FAVC (Frequent consumption of high caloric food) and Age also play crucial roles in the model's predictions. Other notable features include CH2O (Water consumption), FAF (Physical activity frequency), Meal Habits, CALC (Consumption of alcohol), and NCP (Number of main meals), all contributing moderately to the model's predictions. Interestingly, factors like SMOKE, SCC, and FCVC appear to have minimal impact across all obesity classes. This plot also reveals varying feature importance across different obesity classes, potentially indicating complex interactions between features.

## 5 Conclusion

In this comprehensive exploration of machine-learning methods for predicting obesity, several algorithms were evaluated, including LR, SVM, RF, XGBoost, LGBM, CATBoost, and MLP. The models were assessed across seven distinct obesity levels, with RF, XGBoost, LGBM, and CATBoost demonstrating substantial predictive capabilities and high accuracies. However, the proposed hybrid ANN-PSO model outperformed the individual algorithms, achieving the highest accuracy of 92%. Also other performance metrics such as precision, recall, and F1 score were also evaluated, demonstrating the model's superior performance. Additionally, confusion matrices were utilized to understand the classification performance further, and SHAP feature importance analysis was conducted to determine which features had the most significant impact on different obesity classes. The proposed model showcased remarkable robustness, especially in predicting the underrepresented severe obesity categories, and minimized misclassifications, as evidenced by the confusion matrices. This research underscores the potential of hybrid models in enhancing predictive accuracy and managing complex classification tasks with class imbalance. The combination of ANN and PSO techniques proved to be a highly effective strategy, leveraging the strengths of both approaches to achieve superior performance. These findings hold promise for personalized healthcare interventions, showing how important advanced ML methods are for dealing with tough health problems. Accurate obesity classification is crucial for effective risk assessment, treatment planning, and preventive measures, and the proposed model has demonstrated its capability in this regard. This study paves the way for more robust ML solutions in healthcare, ultimately improving patient outcomes. Future work can explore the application of this hybrid approach to other complex classification tasks in healthcare and investigate the impact of different optimization algorithms and network architectures on performance. Additionally, different metaheuristic algorithms, such as Gray Wolf Optimization or Genetic Algorithm, can be investigated to further enhance the predictive accuracy and robustness of obesity prediction models.

## 6 Limitations

The proposed hybrid ANN-PSO model for obesity classification faces several limitations. While removing height from the dataset was a strategic decision to avoid the triviality associated with BMI, this approach may inadvertently exclude valuable information that could enhance the model's accuracy. By focusing on features such as weight, dietary habits, physical activity, genetic markers, and demographic factors, the model aims to address the oversimplification of using BMI alone. However, this adjustment may lead to a potentially diminish the model's overall predictive performance. Additionally, the reliance on PSO for hyperparameter tuning can sometimes result in suboptimal convergence, and the high computational cost of training the model may limit its feasibility for real-time applications. Furthermore, the model's generalizability to diverse populations without retraining remains a concern, which could impact its broader applicability and effectiveness.

## Data Availability

The original contributions presented in the study are included in the article/supplementary material, further inquiries can be directed to the corresponding author.
